# The impacts of Covid-19 on perinatal mental health – Part 2

**Published:** 2022-10-01

**Authors:** O Ibiwoye, JE Hill, G Thomson

**Affiliations:** Liverpool Women’s Hospital; University of Central Lancashire; University of Central Lancashire

## Introduction

A systematic review and meta-analysis undertaken by Tomfohr-Madsen et al. 2021 reports on the prevalence rates of clinically significant anxiety and depression in pregnant women during the pandemic (1). The prevalence estimates suggest that during the pandemic one in four pregnant women have clinically significant depression and one in three pregnant women have clinically significant anxiety. Furthermore, while the evidence indicates that the prevalence of anxiety (but not depression) had significantly increased overtime during the pandemic, there is a need to assess the extent to which the pandemic has been a causative factor.

To this end, the review by Hessami et al 2020 (2). reports changes in anxiety and depression scores in pregnant and postnatal women before and after the pandemic.

### Aim of commentary

To critically appraise the methods used within the review by Hessami et al. 2020 and to consider the implications of the findings within clinical practice (2).

## Methods

The review undertook a multi-database search from date of inception to 5th July 2020. Additional methods to identify suitable literature included reviewing the reference lists of included studies. Only observational studies published in English which reported scores for the Edinburgh Postnatal Depression Scale (EPDS) and State-Trait Anxiety Inventory (STAI) undertaken with pregnant women were included. Title and abstract screening were undertaken by two reviewers independently. However, no description is given for method of data extraction and there was no assessment of methodological bias. A random effects meta-analysis was undertaken to compare mean EPDS during the pandemic and standard means difference (SMD) for both EPDS and STAI scores before and after the pandemic. The difference between studies was assessed using the I^2^ statistic. Assessment of publication bias was undertaken using the Egger’s and Begg’s tests (these tests are commonly used to assess publication bias).

## Results

Eight studies were included in the review. The studies took place in Canada, Italy, China, Turkey, and Greece. There were three meta-analyses. One for the overall mean EPDS score, another for the mean EPDS score pre pandemic vs post pandemic, and the last for the mean STAI score pre pandemic vs post pandemic. All meta-analyses had considerable difference between studies. The mean EPDS scores during the pandemic was 9.84 (95% confidence interval (CI) 8.36 - 11.33, p < .001, I^2^ = 98.7%, 6 studies). The mean score for STAI during the pandemic was not reported. A non-statistically significant moderate effect was found for EPDS score when comparing pre- and post-pandemic scores (SMD = 0.40, 95% CI: −0.05 − 0.86, p = .083; I^2^ = 98.0%, 3 studies). A large statistically significant effect was found for STAI when comparing pre- and post-pandemic scores: 0.82 (95% CI: 0.49 − 1.16, p < .001; I^2^ = 90.2%, 3 studies). The Begg’s and Egger’s test for mean EPDS scores was not statistically significantly different.

## Commentary

Using the Joanna Briggs Institute Critical Appraisal Tools for Systematic Reviews and Meta-analysis, 8 of the 11 domains assessed were judged to be satisfactory for this review (3). The two domains related to the assessment of bias of included studies were unsatisfactory because there was no assessment of bias studies undertaken. This lack of assessment makes it difficult to measure the impact of the possible varying biases of included studies on the overall results. The domain for methods used to combine results was also judged as not satisfactory. According to the Cochrane handbook, “the standardised mean difference (SMD) is used as a summary statistic in meta-analysis when the studies all assess the same outcome but measure it in different ways” (4). In the current review, all the included studies used the same tools. It would have therefore been more appropriate for a simple (not standardised) difference in means meta-analysis to have been undertaken (5).

The primary outcome was the combined mean EPDS score in pregnant and post-partum women. The SMD in EPDS and STAI scores were the secondary outcomes, but the mean STAI score was not reported. Hence, the review has some level of reporting bias. Additionally, for publication bias to be assessed, at least ten studies should be included (6). However, in this review, there were only three and six studies, for anxiety and depression respectively. The levels of differences between studies were also substantial for both outcomes, hence the use of the Egger’s test was not appropriate (7).

Although the results of this review need to be interpreted with caution, they do suggest that increases in perinatal depression and anxiety are related to the pandemic. The EPDS and STAI tools are generally accepted as screening tools (8,9), but wider evidence indicates that they could potentially underestimate the presence of depressive and anxiety symptoms which could have been the case in this review. Women may choose not to identify themselves as depressed or anxious with self-administered tools (10) due to concerns with stigma, fear or being labelled as an inadequate mother, and/or fear of social services involvement (11, 12). According to Button et al. (12), standardised questions may be less personal hence the potential to mask the true picture of how things are. Therefore, apart from prioritising interventions to ameliorate the impact of the pandemic on perinatal mental health, it is imperative to start thinking of more sensitive ways to screen for depression, anxiety, and other mental health disorders that allow women to discuss and disclose their concerns.

It is recommended for future research that the risk factors for new onset mental illness and relapse of established mental health disorders during the pandemic in perinatal women are explored. Additionally, future research should consider exploring the moderating factors for the increased prevalence of anxiety and depression associated with the COVID-19 pandemic. This evidence could then inform evidence-based interventions to ameliorate poor mental health which could ultimately be a basis for primary prevention.

## Key Points

- Increased prevalence of depression and anxiety in the perinatal period may be directly linked to the COVID-19 pandemic
- Screening tools for depression and anxiety may underestimate the impact of the pandemic on perinatal mental health
- There is a need to incorporate more sensitive measures into the screening process for mental illness in the perinatal period
